# Metabolic Syndrome and Insulin Resistance in Romania

**DOI:** 10.3390/ijms26062389

**Published:** 2025-03-07

**Authors:** Adela Gabriela Ştefan, Diana Clenciu, Adina Mitrea, Ionela Mihaela Vladu, Diana Cristina Protasiewicz-Timofticiuc, Maria Magdalena Roşu, Daniela Teodora Maria, Ilie Robert Dinu, Theodora Claudia Gheonea, Beatrice Elena Vladu, Ion Cristian Efrem, Eugen Moţa, Maria Moţa

**Affiliations:** 1 Department of Diabetes, Nutrition and Metabolic Diseases, Calafat Municipal Hospital, 205200 Calafat, Romania; adela.firanescu@yahoo.com; 2 Department of Diabetes, Nutrition and Metabolic Diseases, Faculty of Medicine, University of Medicine and Pharmacy of Craiova, 200349 Craiova, Romania; diana.clenciu@umfcv.ro (D.C.); theodora.gheonea@umfcv.ro (T.C.G.); 3 Department of Diabetes, Nutrition and Metabolic Diseases, Clinical County Emergency Hospital of Craiova, 200642 Craiova, Romania; diana_protasiewicz@yahoo.com; 4 Department of Diabetes, Nutrition and Metabolic Diseases, Faculty of Midwives and Nursing, University of Medicine and Pharmacy of Craiova, 200349 Craiova, Romania; maria.rosu@umfcv.ro; 5 Department of Nephrology, Faculty of Medicine, University of Medicine and Pharmacy of Craiova, 200349 Craiova, Romania; danagiurka@yahoo.com (D.T.M.); roberto_dir@yahoo.com (I.R.D.); 6 Faculty of Medicine, University of Medicine and Pharmacy of Craiova, 200349 Craiova, Romania; beatricevladu75@gmail.com; 7 Department of Internal Medicine—Medical Semiology, Faculty of Dentistry, University of Medicine and Pharmacy of Craiova, 200349 Craiova, Romania; cristian.efrem@umfcv.ro; 8 Doctoral School, University of Medicine and Pharmacy of Craiova, 200349 Craiova, Romania; eugenmota@yahoo.com (E.M.); mmota53@yahoo.com (M.M.)

**Keywords:** metabolic syndrome, insulin resistance, PREDATORR study, Romania

## Abstract

Metabolic syndrome (MetS) represents a huge burden on the health system. This study aimed to investigate the association between MetS and certain indirect insulin resistance (IR) indicators according to gender. The triglyceride–glucose index (TyG), TyG–body mass index (TyG–BMI), the TyG–waist-to-height ratio (TyG–WHtR), TyG–waist circumference (TyG–WC), the triglyceride to high-density-lipoprotein cholesterol index (TG/HDL-c) and recently proposed indicators such as the metabolic score for IR (MetS-IR), TyG–neck circumference (TyG–NC) and the TyG–neck-circumference-to-height ratio (TyG–NHtR) were evaluated in 2594 subjects enrolled in the PREDATORR study. Univariate and multivariate logistic regression was performed to identify the association between MetS and the indirect IR indicators, as well as the risk factors. The participants were divided into two groups, according to gender. Data were analyzed using SPSS version 26.0. TyG, TyG–WC, TyG–NC, TyG–NHtR and TG/HDL-c had higher values in the male group, while TyG–BMI, TyG–WHtR and MetS-IR had approximately equal values in the two studied groups, but also statistically significantly higher values in MetS (+) vs. MetS (−) subjects (*p* < 0.001). For both studied groups, the multivariate logistic regression analysis demonstrated that TyG and MetS-IR were independent predictors for MetS. Both in the female and in the male group, TyG had the largest area under the receiver operating characteristic (AUROC) curve. Thus, in females, the TyG AUROC curve was 0.890; 95% CI 0.873–0.907; *p* < 0.001; cut-off value 8.51, with 81.4% sensitivity and 80.0% specificity. In males, the TyG AUROC curve was 0.880; 95% CI 0.861–0.899; *p* < 0.001; cut-off value 8.69, with 78.5% sensitivity and 84.6% specificity. All of the analyzed indirect IR indicators had statistically significantly higher values in MetS (+) vs. MetS (−) subjects. TyG and MetS-IR are independent predictive factors for MetS, regardless of the subject’s gender.

## 1. Introduction

Metabolic syndrome (MetS) represents one of the most important challenges of the public health system, affecting over a billion adults worldwide, both in developed and developing countries [1,2,3]. MetS involves the association of metabolic abnormalities, characterized by abdominal obesity, insulin resistance (IR), hypertension (HTN) and dyslipidemia, and implies a significant risk of both cardiovascular and all-cause mortality, by increasing the risk of sudden death and the development of cardiovascular diseases, (such as myocardial infarction or stroke) as well as type 2 diabetes mellitus (DM) [1,2,4,5,6,7,8].

In the last decades, there have been dramatic changes in the lifestyle and dietary behavior of the population, involving diets rich in refined carbohydrates and processed foods, and a sedentary lifestyle, as well as a reduced duration of sleep. Sleep deprivation causes suppression of leptin levels and stimulation of ghrelin secretion, compared to a corresponding duration of sleep, which induces appetite stimulation [9,10]. Moreover, studies published in the medical literature reported alcohol consumption and smoking as risk factors for MetS. However, this association is controversial, and the results have been inconclusive [1,11,12]. At the end of the 1980s, Prof. Gerlad M. Reaven and Dr. Norman Kaplan described MetS, and in 1988, Reaven demonstrated the existence of the so-called “Syndrome X”, describing the association between DM, HTN and abdominal obesity, as well as their influence on IR [7,13]. Only a year later, Dr. Norman Kaplan characterized the interaction between abdominal obesity, DM, HTN and hypertriglyceridemia as the “deadly quartet” [7,14].

Since then, numerous definitions of MetS have been proposed, with a consensus being reached in 2009. MetS is currently defined by a set of factors, which represent cardiovascular risk factors per se, being represented by abdominal obesity (increased waist circumference), HTN, hypertriglyceridemia, a reduced level of high-density-lipoprotein cholesterol (HDL-c) and hyperglycemia. To be diagnosed with MetS, a person must meet at least three of these five criteria [15].

MetS involves complex pathogenic pathways, with IR being considered the central mechanism [16,17]. Other important mechanisms are also involved, such as oxidative stress, inflammation and endothelial dysfunction, along with the alteration of lipid metabolism [6,18].

Unfortunately, excess weight and associated complications increasingly affect not only the adult population, but also children and adolescents. Thus, the increase in the prevalence of obesity, type 2 DM and cardiovascular diseases has, in recent years, caused an increase in the prevalence of MetS to 3% among children, 5% among adolescents and over 20% in adults [7,19,20]. In Romania, the prevalence of MetS in adults aged between 20 and 79 years old was 38.5%, while the prevalence of obesity was 31.9%, and the prevalence of being overweight was 34.7% (PREDATORR Study). Moreover, 73.9% of the adult population of Romania had abdominal obesity [21].

Recently, numerous indirect (not based on insulin) biological indicators of IR have been proposed, making the measurement of IR much simpler, namely the triglyceride–glucose index (TyG), TyG–body mass index (TyG–BMI), the TyG–waist-to-height ratio (TyG–WHtR), TyG–waist circumference (TyG–WC), the triglyceride to high-density-lipoprotein cholesterol index, also called the Reaven index (TG/HDL-c), and recently proposed indicators such as the metabolic score for IR (MetS-IR), TyG–neck circumference (TyG–NC) and the TyG–neck-circumference-to-height ratio (TyG–NHtR); all of these have also been studied in patients with MetS [22,23,24,25]. Although the PREDATORR study brought to light data on the prevalence of MetS in our country, as well as some of its components, no data are available regarding the correlation between MetS and the indirect indicators of IR. In this context, we proposed to investigate this association and the differences according to the study participants’ gender.

## 2. Results

In the PREDATORR study, 2728 participants were enrolled, but data were incomplete for 134 of them, so they could not be included in this analysis. Thus, a total of 2594 subjects were eligible for the carried-out analysis, the majority being women (52.6%). The prevalence of MetS, adjusted for age and gender, was reported in another published article, as previously mentioned [21]. The demographic, anthropometric and paraclinical characteristics of the studied group are presented in Table 1.

Both in the female and male groups, the analysis of these characteristics highlighted statistically significant differences between MetS (+) subjects and MetS (−) subjects, as follows: MetS (+) participants presented with an older age, BMI, WC, WHtR, NC, NhtR, %BF, SBP and DBP with significantly increased values. Also, the following paraclinical data demonstrated significantly increased values in subjects with MetS in both genders: FPG, HbA1c, TC, TG and HOMA-IR. HDL-c values were significantly lower in both women and men with MetS.

Regarding sedentarism, a significantly increased percentage was recorded in the group of men with MetS, but in the group of women, a statistically significant difference could not be demonstrated (*p* = 0.425). Reduced sleep duration was more frequent in the group of females with MetS (+), while in the case of males, the results were almost superimposed (*p* = 0.922). Regarding smoking and alcohol consumption, we could not demonstrate a statistically significant difference in the sense of a higher frequency in the case of subjects with MetS, as presented in Table 1.

We calculated and compared the indirect IR indicators according to the two studied groups. It is extremely important that all analyzed indicators, regardless of gender, had statistically significantly higher values in the case of MetS (+) vs. MetS (−) subjects (*p* < 0.001), as can be seen in Table 2.

Moreover, using the Spearman’s correlation coefficient, we observed that the indirect indicators of IR positively correlate with each other in both genders, as is evidenced in Table 3 and Table 4.

Univariate logistic regression analysis highlighted, both in the case of females and males, that with the exception of LDL-c, which was associated with MetS only in females, all of the other parameters investigated were associated with MetS in both genders. The established limit for the analysis of WHtR and NhtR represents the cut-off of the respective variables (Table 5).

Using the multivariate logistic regression analysis adjusted for age, we demonstrated that TyG and MetS-IR were independent predictors for MetS in males and females alike. Furthermore, we could observe that TyG–BMI and TG/HDL-c have lower chances of being associated with MetS, regardless of gender (*p* < 0.001). In the case of the other studied indicators, statistical significance was not reached (Table 6).

**Figure 1:**
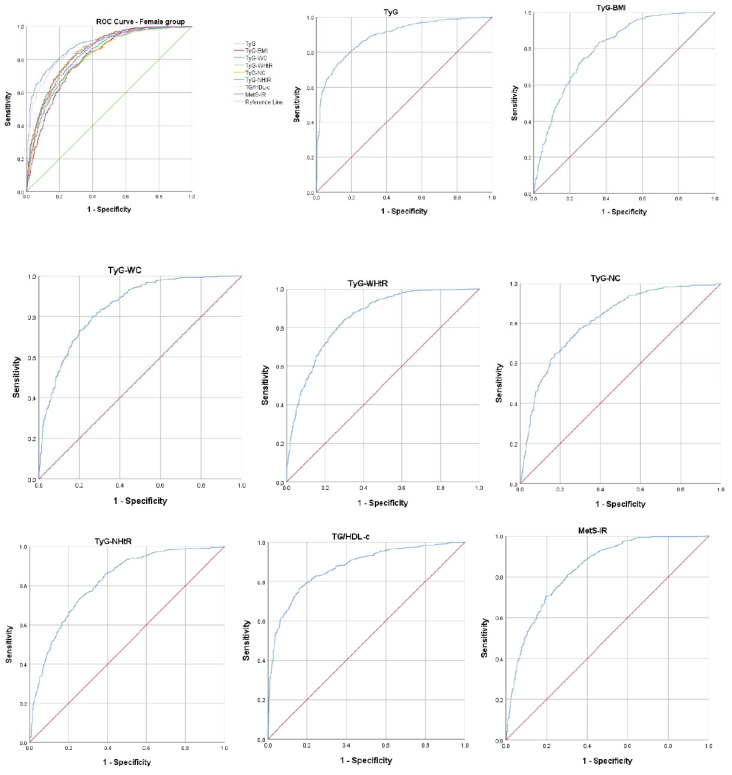
The ROC curve analysis for the indirect IR indicators in females. ROC: receiver operating characteristic; TyG: triglyceride–glucose; TyG–BMI: TyG–body mass index; TyG–WC: TyG–waist circumference; TyG–WHtR: TyG–waist-to-height ratio; TyG–NC: TyG-neck circumference; TyG–NHtR: TyG-neck-circumference-to-height ratio; TG/HDL-c: triglyceride to high-density-lipoprotein cholesterol; MetS-IR: metabolic score for insulin resistance.

Regarding the ROC curve analysis, for both genders, TyG had the highest AUROC curve. Thus, in the case of female subjects, the TyG AUROC curve was 0.890, with 95% CI 0.873–0.907, and *p* < 0.001; the cut-off value was 8.51, with 81.4% sensitivity and 80.0% specificity. The next indicator with the highest AUROC curve was TG/HDL-c (AUROC curve 0.872; 95% CI 0.853–0.891); *p* < 0.001; cut-off value 2.05, presenting 80.4% sensitivity and 79.0% specificity. Data on all analyzed indirect IR indicators are presented in Figure 1 and Table 7.

**Figure 2:**
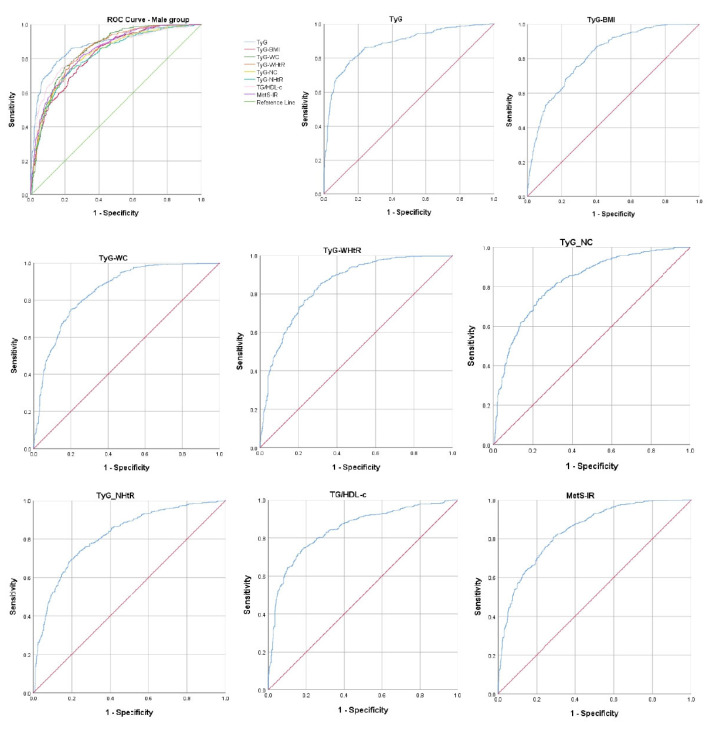
The ROC curve analysis for the indirect IR indicators in males. ROC: receiver operating characteristic; TyG: triglyceride–glucose; TyG–BMI: TyG–body mass index; TyG–WC: TyG–waist circumference; TyG–WHtR: TyG–waist-to-height ratio; TyG–NC: TyG-neck circumference; TyG–NHtR: TyG-neck-circumference-to-height ratio; TG/HDL-c: triglyceride to high-density-lipoprotein cholesterol; MetS-IR: metabolic score for insulin resistance.

In the case of male study participants, the TyG AUROC curve recorded the value 0.880, with 95% CI 0.861–0.899 and *p* < 0.001. The cut-off value was 8.69, the sensitivity 78.5%, and the specificity 84.6%. After TyG, the next indicator with the highest AUROC curve was TyG–WC (AUROC curve 0.851; 95% CI 0.831–0.872; *p* < 0.001; cut-off value 895.51; 74.8% sensitivity; 80.3% specificity). The data regarding indirect indicators of IR in males are presented in Figure 2 and Table 8.

**Figure 3:**
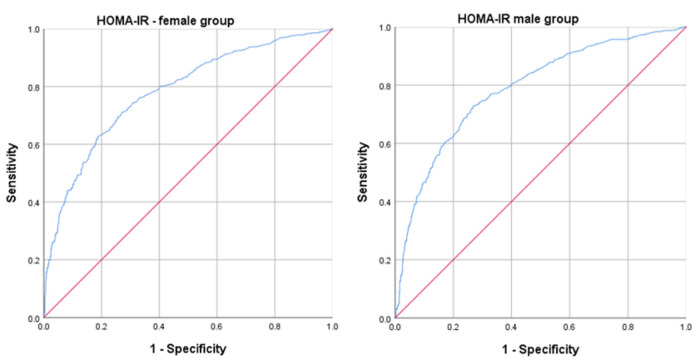
The ROC curve analysis for HOMA-IR in the female group and the male group. ROC: receiver operating characteristic; HOMA-IR: homeostatic model assessment for IR. Blue line: AUROC values; Red line: Reference line.

Compared to the values obtained regarding the indirect IR indicators, the HOMA-IR presented the following characteristics in the female group: AUROC curve 0.781; standard error 0.013; 95% CI 0.756–0.805; *p* < 0.001; cut-off value 2.125; sensitivity 71.0%; specificity 73.0%. In the male group, the HOMA-IR demonstrated the following: AUROC curve 0.787; standard error 0.013; 95% CI 0.762–0.812; *p* < 0.001; cut-off value 2.085; sensitivity 72.9%; specificity 73.1% (Figure 3).

## 3. Discussion

As we mentioned, in Romania, the prevalence of MetS was 38.5%, increasing with age and being more frequent in male participants, compared to female participants. These data were highlighted in a previously published article [21]. Recently, the relationship between indirect indicators of IR and MetS has become a topic of interest for researchers [26,27,28]. These indicators are useful in the diagnosis of MetS [16,24,29,30,31,32], and the diagnostic level and value show differences depending on the gender of the subjects [16], a fact confirmed by our study.

Abbasi and Reaven published an article in 2011, in which the correlation between TyG and MetS was analyzed and proven [33]. TyG proved to be the best predictor of MetS in overweight and obese subjects, but also in normal-weight individuals, which is remarkable since early identification and intervention of the risk factors can be performed [16,24,25,34]. The fact that TyG represents an important indicator for MetS screening, being sensitive and specific for this condition, was also proven in a systematic review and meta-analysis published by Nabipoorashrafi et al. [35]. These results were also found in a cross-sectional study by Zhang et al., highlighting that TyG is an independent risk factor for MetS, but also an important indicator that can be used for MetS screening [16].

According to the results of our study, out of all of the indirect IR indicators analyzed, TyG presents the highest diagnostic accuracy, confirming results published by other authors [24,32]. Moreover, similar to our study, other authors evidenced the usefulness and importance of using indirect IR indicators. Thus, TyG presented a higher AUROC curve compared to the HOMA-IR, demonstrating that it has a superior capacity to predict MetS [22,24,30,36]. The same higher predictive value of TyG over the HOMA-IR was reported in a prospective study from 2022 in over 6000 subjects [37]. Furthermore, in a recent study of over 9000 subjects, TyG demonstrated predictive value for type 2 diabetes mellitus at the cut-off value 8.9, with a sensitivity of 72.3% and a specificity of 79.1% [38]. In disagreement with our results, which demonstrate that TyG has the highest diagnostic accuracy of MetS, compared to combinations of TyG and anthropometric indices, Lim et al. published data indicating TyG–BMI as the indicator with the best prediction of IR, compared to TyG, TyG–WC and TyG–WHtR [31]. Similarly, Raimi et al. conducted a study that places TyG after TyG–WHtR, TyG–WC and TyG–BMI in terms of the AUROC curve for the diagnosis of MetS [29]. The explanation can be represented by the characteristics of the study participants, such as ethnic differences, as well as BMI [29,31].

Our study results reveal the fact that both TyG–NC and TyG–NHtR, although they have comparatively lower AUROC curves, are useful in the diagnosis of MetS, with the importance of these results being amplified by the fact that NC represents an important indicator for cardiovascular risk and MetS [39,40]. Similar results were described by Mirr et al., which further highlight the need for additional observations for the application of these indirect indicators of IR in clinical practice [24].

Because MetS is closely related to lipid abnormalities in terms of its diagnostic criteria, TG/HDL-c achieves the second highest AUROC curve in our study in the case of female subjects and the third highest AUROC curve in the case of male subjects. Similarly, Mirr et al. described this indicator as having the highest AUROC curve after TyG [24]. Contrary to our results, the study of Zhang et al. [16] reported that TG/HDL-c had one of the lowest AUROC curve among the analyzed IR indicators. Moreover, TG/HDL-c was not reported as an independent risk factor for women with MetS [16], unlike the results obtained in our study.

Bello-Chavolla et al. demonstrated for the first time that MetS-IR can be used for cardiometabolic risk assessment and MetS screening [34,41]. However, similar results to our study were published by other authors, who found that MetS-IR did not have a higher diagnostic accuracy of MetS compared to other IR indicators, in both genders [16,24]. Moreover, Zhang et al. [16] demonstrated that MetS-IR was an independent risk factor for women and men with MetS; these results were also confirmed in a recent study conducted in 250 adolescents aged 13–18 years old [42]. In this research, the cut-off value identified for MetS-IR, predictive for MetS, was 46.53, having a specificity of 75.76% and a sensitivity of 64.24% [42]. In the present study, TyG and MetS-IR were independent predictors for MetS, regardless of the subject’s gender.

The limitations of this study must be also considered and are represented by the fact that the study was conducted in a specific geographic region, and the results cannot be extrapolated to other populations, except for those with similar characteristics. Another limitation is represented by the fact that this is a cross-sectional study, and MetS and the analyzed factors coexist; therefore, we cannot establish a cause–effect relationship. In addition, this study managed to compare the indirect IR indicators with only the HOMA-IR, not with other indicators used for the diagnosis of MetS. The small number of subjects in different analyzed subgroups may represent an explanation for the wide confidence intervals in the logistic regression analysis, this being another limitation of our study. Last, but not least, the major cardiovascular risk factors were not analyzed, such as metabolic-dysfunction-associated steatotic liver disease (MASLD) and chronic kidney disease (CKD), elements that have recently been identified as being diagnostic criteria for MetS [7,43]. MASLD is considered to be the hepatic manifestation of MetS, given the fact that they share numerous cardiometabolic risk factors, such as obesity, IR, HTN, atherogenic dyslipidemia, type 2 DM or prediabetes [7,44,45]. MASLD is characterized by an increased hepatic fat content, not being caused by significant alcohol consumption or another secondary cause of steatosis; recent data has suggested that hepatic fat accumulation may influence the transformation of metabolically healthy obesity into metabolically unhealthy obesity, thus exposing the person to an increased cardiometabolic risk. However, MASLD is not a diagnostic criterion for MetS [7,46,47]. MASLD can be evaluated using liver ultrasound or transaminases, or a combination of these markers. In a study conducted by our team in 2022 [26], we highlighted the usefulness of insulin resistance biomarkers as predictors for MASLD in patients with type 2 diabetes. Furthermore, in two other studies [27,28], we demonstrated that TyG is a useful tool for the evaluation of MASLD in patients with MetS, as it correlates with the degree of liver steatosis described using liver biopsy. In light of this, the study of transaminases in our study population would have brought important information regarding this association. However, the study design of the PREDATORR study did not include the measurement of these biomarkers, which is a limitation for the present analysis.

This study also presents strong points represented mainly by the calculation of the sample size, but also by the standardized measurements of the anthropometric and paraclinical characteristics, as well as the establishment of diagnoses according to the recommendations of the current guidelines.

Although studies have been carried out in various regions and on various populations, this topic has not been sufficiently investigated, as complex studies are needed on large groups of subjects to identify a uniform diagnostic indicator of MetS. What is more, identifying early markers of MetS, with low costs, that can be easily calculated based on routine laboratory investigations, is particularly useful in the situation of limited resources, which is the case in many health care systems around the world.

## 4. Materials and Methods

### 4.1. Participants

The PREvalence of DiAbeTes mellitus, prediabetes, overweight, Obesity, dyslipidemia, hyperuricemia and chRonic kidney disease in Romania (PREDATORR) study (EudraCT number: 2012-004803-12) took place in Romania, during 2012–2014. This was a cross-sectional, population-based study. It aimed to determine the prevalence of DM, prediabetes, being overweight and obesity, dyslipidemia, hyperuricemia and CKD in the adult population. The design of this study has been described in previously published articles [21,48]. The subjects were enrolled through a random computerized selection from the databases of 101 general practitioners from the National Health Insurance Company database, also randomly enrolled. This was carried out equally for the eight historical regions of Romania. In order to produce a representative sample for the adult population in our country, based on the 2002 Romanian Census, a total of 2728 participants who met the following inclusion criteria were included in the study: age between 20 and 79 years, born in Romania, living in Romania for the last 10 years, included in the list of a general practitioner, not pregnant and not lactating. Written informed consent was obtained from all the participants before being subjected to the specific procedures of the PREDATORR study. In addition, the study was approved by the Romanian National Ethics Committee (approval code 4064 from 12 December 2012) and was carried out in accordance with the World Medical Association Declaration of Helsinki—Ethical Principles for Medical Research Involving Human Participants, and the applicable standards of the International Conference on Harmonization (ICH)/Good Clinical Practice (GCP).

### 4.2. Anamnestic, Lifestyle and Socio Demographic Data

The interviewers used a questionnaire to collect information on socio-demographic characteristics (age, gender, education level, marital status), lifestyle (physical activity, sleep duration, alcohol consumption, diet, smoking), as well as medical history (DM, HTN, dyslipidemia, obesity, cardiovascular diseases, etc., along with current lipid-lowering, antihypertensive and antidiabetic treatment). The education level of the participants was classified as low (primary school, secondary school) or high (college, high school, university). Subjects who performed physical activity less than 4 days per week were considered sedentary. Reduced sleep duration was defined in participants who slept less than 7 h per night. Regarding alcohol consumption, subjects who declared that they had not consumed alcohol in the last month were considered non-drinkers. Smoking data were collected through a self-administered questionnaire. Subjects were therefore categorized by smoking status into non-smokers (never smoked), current smokers (smoked more than one cigarette per day, occasionally, or daily and did not quit), and former smokers (who have quit smoking).

### 4.3. Clinical and Biochemical Data

The clinical examination consisted of recording anthropometric data (height, weight, and waist, hip and neck circumference), as well as systolic and diastolic blood pressure, using standard procedures. BMI was calculated according to the formula BMI = weight (kilograms)/height squared (meters), and the results were classified according to the criteria of the World Health Organization. Being overweight was defined as having a BMI between 25 and 29.9 kg/m^2^, and subjects with a BMI ≥ 30 kg/m^2^ were classified as obese. Abdominal obesity was defined as a waist circumference (WC) of ≥80 cm for women and ≥94 cm for men. The ratio between waist circumference and height (WHtR) was calculated according to the following formula: waist circumference (cm)/height (cm), with a value ≥0.5 being an indicator of abdominal obesity. The ratio between neck circumference and height (NhtR) was calculated according to the following formula: neck circumference (cm)/height (cm) [49]. High blood pressure was defined as a SBP ≥ 130 mmHg and/or DBP ≥ 85 mmHg and/or personal history of HTN and/or home antihypertensive treatment [15]. We also calculated body fat percentage (%BF), according to the Deurenberg equation: %BF = 1.2 × BMI (kg/m^2^) + 0.23 × age (years) − 10.8 × gender (female = 0, male = 1) − 5.4 [50]. The homeostatic model assessment for IR (HOMA-IR) was estimated using the equation proposed by Matthews et al. [51].

The blood samples were collected while fasting, and biochemical analyses were performed according to standardized procedures. Enzymatic methods were used to determine fasting plasma glucose (FPG), total cholesterol (TC), HDL-c, low-density-lipoprotein cholesterol (LDL-c) and triglycerides (TG). Hyperglycemia was defined by an FPG value ≥ 100 mg/dL and/or glucose-lowering medication at home. Hypertriglyceridemia was defined as a TG value ≥ 150 mg/dL or if the subjects were taking drug treatment for hypertriglyceridemia. Hypo-HDL cholesterolemia was considered when HDL-c levels were <40 mg/dL in men and <50 mg/dL in women and/or if participants received treatment for low HDL-c.

The indirect indicators of IR were calculated using the following formulas: TyG = Ln (fasting TG × FPG/2), TyG–BMI = TyG × BMI, TyG–WHtR = TyG × WHtR, TyG–WC = TyG × WC, TG/HDL-c = fasting TG/fasting HDL-c, MetS-IR = (Ln (2 × FPG + fasting TG) × BMI)/(Ln (HDL-c)), TyG–NC = TyG × NC, TyG–NHtR = TyG × NHtR; where Ln represents natural logarithm [24,25,29,34,41].

MetS was defined according to the Harmonization definition, 2009, if the subjects met three out of the five diagnostic criteria presented in Table 9 [15].

### 4.4. Statistical Analysis

The PREDATORR study used a cluster sampling design in order to select the subjects who participated in the study. We used the Kolmogorov–Smirnov test to identify the distribution of continuous variables. Data with a Gaussian distribution were presented as the mean ± standard deviation (SD), and data that did not have a normal distribution were presented as the median and interquartile range (IQR). To determine the significance of the differences between the groups, the Student’s *t*-test was used to compare the means, and the Mann–Whitney U test was used to compare the medians. Categorical variables were analyzed using the chi-square test. The cut-off value was evaluated by analyzing the area under the receiver operating characteristic (AUROC) curve. The indirect indicators of IR were correlated using the Spearman’s correlation coefficient. Univariate and multivariate logistic regression was performed, with the aim of identifying the relationships between MetS and indirect indicators of IR, as well as risk factors, providing the odds ratio (OR) with 95% confidence intervals (95% CI). The analyses were performed according to gender. The obtained results were considered to be statistically significant if the recorded *p*-value was < 0.05. Data were analyzed using the Statistical Package for the Social Sciences (SPSS) version 26.0 (SPSS Inc., Chicago, IL, USA).

## 5. Conclusions

The values of the indicators TyG, TyG–WC, TyG–NC, TyG–NHtR and TG/HDL-c had higher values in the male group, while TyG–BMI, TyG–WHtR and MetS-IR had similar values in the two studied groups. Moreover, all of the analyzed indirect indicators of IR, regardless of gender, had statistically significantly higher values in the case of MetS (+) vs. MetS (−) subjects. Among these indicators, TyG demonstrated the highest AUROC curve, both in women and in men. TyG and MetS-IR were independent predictive factors for MetS, regardless of the gender of the study participants. In addition, TyG showed a higher AUROC curve even compared to the HOMA-IR, demonstrating that it has a superior capacity to predict MetS, which is extremely important in daily clinical practice. These results can be useful for the diagnosis of MetS, but also for the implementation of individualized prevention measures. It is extremely important to identify people with MetS early through screening to optimize the management of risk factors and to implement lifestyle changes.

## Figures and Tables

**Table 1:** Demographic, anthropometric and paraclinical characteristics of the study population.

Characteristics	Female Group			Male Group		
	MetS (+)	MetS (−)	*p*-Value	MetS (+)	MetS (−)	*p*-Value
Participants, no.	595	782		590	627	
Age (years)	63 (13)	53 (22)	<0.001	61 (15)	57 (24)	<0.001
BMI (kg/m^2^)	30.8 (6.9)	26 (7.6)	<0.001	29.4 (5.5)	26.3 (5.4)	<0.001
WC (cm)	101.5 (16)	88 (20)	<0.001	106 (13)	97.2 (16)	<0.001
WHtR	0.63 (0.10)	0.54 (0.13)	<0.001	0.61 (0.08)	0.56 (0.09)	<0.001
NC (cm)	37 (4)	34 (5)	<0.001	42 (4)	40 (5)	<0.001
NhtR	0.23 (0.03)	0.21 (0.03)	<0.001	0.23 (0.03)	0.22 (0.03)	<0.001
SBP (mmHg)	140 (25)	127 (28)	<0.001	145 (28)	133 (29)	<0.001
DBP (mmHg)	81 (16)	76 (15)	<0.001	84 (15)	79 (17)	<0.001
FPG (mg/dL)	96.17 (31)	80 (14)	<0.001	99 (32)	81.99 (16)	<0.001
HbA1c (%)	5.80 (0.9)	5.38 (0.5)	<0.001	5.70 (1.0)	5.40 (0.4)	<0.001
TC (mg/dL)	212 (75)	204.5 (59)	0.006	204 (64)	200 (57)	0.014
HDL-c (mg/dL)	48 (14)	62 (17)	<0.001	42 (14)	53.36 (17)	<0.001
LDL-c (mg/dL)	133 (64)	124 (50)	<0.001	123 (54)	121.34 (50)	0.638
TG (mg/dL)	153.57 (79)	88 (49)	<0.001	172 (110)	98.94 (53)	<0.001
%BF	45.68 (8.28)	38.66 (11.66)	<0.001	32.94 (7.37)	28.54 (8.39)	<0.001
HOMA-IR	4.01 (2.72)	1.53 (1.17)	<0.001	4.07 (3.04)	1.85 (1.21)	<0.001
Marital status (%)						
Married	62.5	69.1	<0.001	86.7	78.9	<0.001
Single	4.1	9.0		3.2	11.8	
Divorced	6.1	7.7		5.1	4.3	
Widowed	27.3	14.2		5.0	4.9	
High educational level (%)	55.7	67.0	<0.001	59.1	59.9	0.772
Sedentarism (%)	18.1	19.8	0.425	19.9	15.6	0.047
Reduced sleep duration (%)	43.2	32.8	<0.001	28.5	28.8	0.922
Alcohol consumption (%)	34.6	36.2	0.550	76.0	79.8	0.107
Smoking (%)						
Daily smokers	12.9	15.1	0.021	19.5	22.1	0.125
Occasional smokers	1.3	3.7		2.6	4.2	
Former smokers	16.9	17.1		43.7	38.3	
Non-smokers	68.9	64.1		34.2	35.4	

MetS: metabolic syndrome; BMI: body mass index; WC: waist circumference; WHtR: waist-to-height ratio; NC: neck circumference; NhtR: neck-circumference-to-height ratio; SBP: systolic blood pressure; DBP: diastolic blood pressure; FPG: fasting plasma glucose; HbA1c: glycated hemoglobin; TC: total cholesterol; HDL-c: high-density-lipoprotein cholesterol; LDL-c: low-density-lipoprotein cholesterol; TG: triglycerides; %BF: body fat percentage; HOMA-IR: homeostatic model assessment for IR. Continuous variables with abnormal distribution are presented as median (IQR).

**Table 2:** Indirect IR indicators according to gender and MetS.

IR Indicators	Female Group			Male Group		
	MetS (+)	MetS (−)	*p*-Value	MetS (+)	MetS (−)	*p*-Value
TyG	8.90 [0.64]	8.18 (0.60)	<0.001	9.05 (0.72)	8.30 (0.57)	<0.001
TyG–BMI	276.35 (64.57)	213.82 (67.02)	<0.001	268.50 (59.93)	217.41 (50.68)	<0.001
TyG–WC	912.20 ± 128.78	724.17 ± 131.97	<0.001	982.33 ± 135.99	810.24 ± 122.20	<0.001
TyG–WHtR	5.70 ± 0.81	4.49 ± 0.84	<0.001	5.65 ± 0.80	4.66 ± 0.69	<0.001
TyG–NC	329.12 (52.59)	281.60 (49.68)	<0.001	378.90 (59.30)	328.60 (46.33)	<0.001
TyG–NHtR	2.04 (0.35)	1.75 (0.32)	<0.001	2.18 (0.34)	1.89 (0.28)	<0.001
TG/HDL-c	3.08 (2.04)	1.40 (0.94)	<0.001	4.17 (3.62)	1.83 (1.37)	<0.001
MetS-IR	47.30 (12.44)	34.87 (11.00)	<0.001	47.36 (11.95)	37.23 (9.24)	<0.001

IR: insulin resistance; MetS: metabolic syndrome; TyG: triglyceride–glucose; TyG–BMI: TyG–body mass index; TyG–WC: TyG–waist circumference; TyG–WHtR: TyG–waist-to-height ratio; TyG–NC: TyG-neck circumference; TyG–NHtR: TyG-neck-circumference-to-height ratio; TG/HDL-c: triglyceride to high-density-lipoprotein cholesterol; MetS-IR: metabolic score for insulin resistance. Continuous variables with normal distribution are presented as means ± standard deviation and those with abnormal distribution are presented as median [IQR].

**Table 3:** Correlation between the indirect IR indicators in females.

IR Indicators	TyG	TyG–BMI	TyG–WC	TyG–WHtR	TyG–NC	TyG–NHtR	TG/HDL-c	MetS-IR
TyG	1							
TyG–BMI	0.611 *	1						
TyG–WC	0.682 *	0.874 *	1					
TyG–WHtR	0.680 *	0.883 *	0.975 *	1				
TyG–NC	0.713 *	0.727 *	0.779 *	0.746 *	1			
TyG–NHtR	0.714 *	0.746 *	0.757 *	0.787 *	0.953 *	1		
TG/HDL-c	0.873 *	0.543 *	0.605 *	0.594 *	0.623 *	0.612 *	1	
MetS-IR	0.606 *	0.971 *	0.865 *	0.868 *	0.715 *	0.728 *	0.615 *	1

IR: insulin resistance; TyG: triglyceride–glucose; TyG–BMI: TyG–body mass index; TyG–WC: TyG–waist circumference; TyG–WHtR: TyG–waist-to-height ratio; TyG–NC: TyG-neck circumference; TyG–NHtR: TyG-neck-circumference-to-height ratio; TG/HDL-c: triglyceride to high-density-lipoprotein cholesterol; MetS-IR: metabolic score for insulin resistance. * *p* < 0.001.

**Table 4:** Correlation between the indirect IR indicators in males.

IR Indicators	TyG	TyG–BMI	TyG–WC	TyG–WHtR	TyG–NC	TyG–NHtR	TG/HDL-c	MetS-IR
TyG	1							
TyG–BMI	0.619 *	1						
TyG–WC	0.678 *	0.818 *	1					
TyG–WHtR	0.674 *	0.824 *	0.969 *	1				
TyG–NC	0.588 *	0.593 *	0.621 *	0.600 *	1			
TyG–NHtR	0.578 *	0.595 *	0.588 *	0.626 *	0.971 *	1		
TG/HDL-c	0.757 *	0.416 *	0.453 *	0.443 *	0.418 *	0.404 *	1	
MetS-IR	0.629 *	0.954 *	0.795 *	0.791 *	0.582 *	0.576 *	0.540 *	1

IR: insulin resistance; TyG: triglyceride–glucose; TyG–BMI: TyG–body mass index; TyG–WC: TyG–waist circumference; TyG–WHtR: TyG–waist-to-height ratio; TyG–NC: TyG-neck circumference; TyG–NHtR: TyG-neck-circumference-to-height ratio; TG/HDL-c: triglyceride to high-density-lipoprotein cholesterol; MetS-IR: metabolic score for insulin resistance. * *p* < 0.001.

**Table 5:** The univariate logistic regression analysis—variables associated with MetS.

Variables	Female Group			Male Group		
	OR	95% CI	*p*-Value	OR	95% CI	*p*-Value
Age (years)	1.057	1.047–1.066	<0.001	1.022	1.013–1.030	<0.001
BMI (kg/m^2^)	1.135	1.111–1.159	<0.001	1.202	1.165–1.239	<0.001
WC (cm)	1.076	1.066–1.086	<0.001	1.070	1.058–1.082	<0.001
WHtR (≧0.5852)	5.868	4.634–7.430	<0.001	4.116	3.253–5.209	<0.001
NC (cm)	1.162	1.126–1.198	<0.001	1.101	1.069–1.134	<0.001
NhtR (≧0.2293)	3.099	2.471–3.886	<0.001	2.888	2.285–3.650	<0.001
SBP (mmHg)	1.030	1.024–1.035	<0.001	1.024	1.018–1.030	<0.001
DBP (mmHg)	1.034	1.024–1.044	<0.001	1.030	1.020–1.039	<0.001
FPG (mg/dL)	1.069	1.059–1.079	<0.001	1.054	1.045–1.062	<0.001
HbA1c (%)	6.834	5.189–9.000	<0.001	4.247	3.302–5.463	<0.001
TC (mg/dL)	1.003	1.001–1.005	0.007	1.003	1.001–1.006	0.008
HDL-c (mg/dL)	0.916	0.905–0.926	<0.001	0.926	0.916–0.937	<0.001
LDL-c (mg/dL)	1.006	1.003–1.008	<0.001	1.001	0.998–1.003	0.685
TG (mg/dL)	1.028	1.025–1.032	<0.001	1.019	1.017–1.022	<0.001
% BF	1.124	1.106–1.143	<0.001	1.141	1.117–1.166	<0.001
HOMA-IR	1.821	1.667–1.988	<0.001	1.704	1.564–1.855	<0.001
TyG	52.985	34.600–81.139	<0.001	29.123	19.900–42.622	<0.001
TyG–BMI	1.021	1.019–1.024	<0.001	1.032	1.028–1.036	<0.001
TyG–WC	1.011	1.010–1.012	<0.001	1.012	1.011–1.013	<0.001
TyG–WHtR	5.820	4.811–7.040	<0.001	7.363	5.808–9.335	<0.001
TyG–NC	1.031	1.027–1.035	<0.001	1.027	1.023–1.030	<0.001
TyG–NHtR	145.452	80.931–261.410	<0.001	79.122	44.325–141.234	<0.001
TG/HDL-c	4.484	3.792–5.302	<0.001	2.135	1.933–2.358	<0.001
MetS-IR	1.149	1.131–1.168	<0.001	1.204	1.178–1.231	<0.001

OR: odds ratio; CI: confidence interval; BMI: body mass index; WC: waist circumference; WHtR: waist-to-height ratio; NC: neck circumference; NhtR: neck-circumference-to-height ratio; SBP: systolic blood pressure; DBP: diastolic blood pressure; FPG: fasting plasma glucose; HbA1c: glycated hemoglobin; TC: total cholesterol; HDL-c: high-density-lipoprotein cholesterol; LDL-c: low-density-lipoprotein cholesterol; TG: triglycerides; %BF: body fat percentage; HOMA-IR: homeostatic model assessment for IR; TyG: triglyceride–glucose; TyG–BMI: TyG–body mass index; TyG–WC: TyG–waist circumference; TyG–WHtR: TyG–waist-to-height ratio; TyG–NC: TyG-neck circumference; TyG–NHtR: TyG-neck-circumference-to-height ratio; TG/HDL-c: triglyceride to high-density-lipoprotein cholesterol; MetS-IR: metabolic score for insulin resistance.

**Table 6:** The multivariate logistic regression analysis adjusted for age.

Variables	Female Group			Male Group		
	OR	95% CI	*p*-Value	OR	95% CI	*p*-Value
TyG	86.426	40.023–186.631	<0.001	40.242	21.488–75.365	<0.001
TyG–BMI	0.898	0.880–0.915	<0.001	0.944	0.930–0.959	<0.001
TyG–WC	1.002	0.961–1.044	0.933	1.006	0.973–1.040	0.728
TyG–WHtR	2.377	0.003–1836.364	0.799	0.662	0.002–202.588	0.888
TyG–NC	0.999	0.893–1.119	0.990	1.002	0.921–1.089	0.970
TyG–NHtR	0.618	0.000–46,825,902.32	0.959	0.896	0.000–1,981,435.421	0.988
TG/HDL-c	0.677	0.561–0.818	<0.001	0.793	0.720–0.874	<0.001
MetS-IR	1.881	1.684–2.101	<0.001	1.448	1.336–1.569	<0.001

OR: odds ratio; CI: confidence interval; TyG: triglyceride–glucose; TyG–BMI: TyG–body mass index; TyG–WC: TyG–waist circumference; TyG–WHtR: TyG–waist-to-height ratio; TyG–NC: TyG-neck circumference; TyG–NHtR: TyG-neck-circumference-to-height ratio; TG/HDL-c: triglyceride to high-density-lipoprotein cholesterol; MetS-IR: metabolic score for insulin resistance.

**Table 7:** The ROC curve analysis for the indirect IR indicators in females.

IR Indicators	AUROC Curve	Standard Error	95% CI	*p*-Value	Cut-Off Value	Sensitivity (%)	Specificity (%)
TyG	0.890	0.009	0.873–0.907	<0.001	8.51	81.4%	80.0%
TyG–BMI	0.806	0.011	0.784–0.828	<0.001	244.52	74.4%	86.0%
TyG–WC	0.848	0.010	0.829–0.868	<0.001	805.72	79.5%	74.0%
TyG–WHtR	0.849	0.010	0.829–0.868	<0.001	5.06	79.4%	74.2%
TyG–NC	0.814	0.011	0.791–0.836	<0.001	306.46	72.4%	74.9%
TyG–NHtR	0.817	0.011	0.795–0.839	<0.001	1.89	74.3%	73.2%
TG/HDL-c	0.872	0.010	0.853–0.891	<0.001	2.05	80.4%	79.0%
MetS-IR	0.836	0.010	0.815–0.857	<0.001	41.02	74.3%	75.8%

IR: insulin resistance; ROC: receiver operating characteristic; AUC: area under the ROC curve; CI: confidence interval; TyG: triglyceride–glucose; TyG–BMI: TyG–body mass index; TyG–WC: TyG–waist circumference; TyG–WHtR: TyG–waist-to-height ratio; TyG–NC: TyG-neck circumference; TyG–NHtR: TyG-neck-circumference-to-height ratio; TG/HDL-c: triglyceride to high-density-lipoprotein cholesterol; MetS-IR: metabolic score for insulin resistance.

**Table 8:** The ROC curve analysis for the indirect IR indicators in males.

IR Indicators	AUROC Curve	Standard Error	95% CI	*p*-Value	Cut-Off Value	Sensitivity (%)	Specificity (%)
TyG	0.880	0.010	0.861–0.899	<0.001	8.69	78.5%	84.6%
TyG–BMI	0.818	0.012	0.795–0.840	<0.001	241.54	75.3%	71.7%
TyG–WC	0.851	0.011	0.831–0.872	<0.001	895.51	74.8%	80.3%
TyG–WHtR	0.845	0.011	0.824–0.866	<0.001	5.07	76.9%	76.6%
TyG–NC	0.823	0.012	0.800–0.846	<0.001	352.06	75.3%	75.5%
TyG–NHtR	0.815	0.012	0.792–0.838	<0.001	2.03	73.8%	76.0%
TG/HDL-c	0.845	0.011	0.824–0.867	<0.001	2.87	74.6%	81.5%
MetS-IR	0.840	0.011	0.819–0.862	<0.001	41.07	80.5%	71.2%

IR: insulin resistance; ROC: receiver operating characteristic; AUC: area under the ROC curve; CI: confidence interval; TyG: triglyceride-glucose; TyG–BMI: TyG–body mass index; TyG–WC: TyG–waist circumference; TyG–WHtR: TyG–waist-to-height ratio; TyG–NC: TyG-neck circumference; TyG–NHtR: TyG-neck-circumference-to-height ratio; TG/HDL-c: triglyceride to high-density-lipoprotein cholesterol; MetS-IR: metabolic score for insulin resistance.

**Table 9:** Criteria for clinical diagnosis of MetS (adapted from [15]).

Measure	Categorical Cut Points
Elevated waist circumference *	Population- and country-specific definitions (Europid: Men ≥ 94 cm, Women ≥ 80 cm)
Elevated triglycerides (or drug treatment for elevated triglycerides ^†^)	≥150 mg/dL
Reduced HDL-c (or drug treatment for reduced HDL-c ^†^)	<40 mg/dL in males; <50 mg/dL in females
Elevated blood pressure (or antihypertensive drug treatment in a patient with a history of hypertension)	Systolic ≥ 130 and/or diastolic ≥ 85 mmHg
Elevated fasting glucose ^‡^ (or drug treatment of elevated glucose)	≥100 mg/dL

* It is recommended that the IDF or AHA/NHLBI cut-off points are used for people of European origin until more data are available. ^†^ The most commonly used drugs for elevated triglycerides and reduced HDL-c are fibrates and nicotinic acid. A patient taking 1 of these drugs can be presumed to have high triglycerides and low HDL-c. High-dose omega-3 fatty acids presumes high triglycerides. ^‡^ Most patients with type 2 diabetes mellitus will have metabolic syndrome by the proposed criteria.

## Data Availability

All of the data are contained in the manuscript.

## References

[1] Jamali Z., Ayoobi F., Jalali Z., Bidaki R., Lotfi M.A., Esmaeili-Nadimi A., Khalili P. (2024). Metabolic syndrome: A population-based study of prevalence and risk factors. Sci. Rep..

[2] Badawy M., Elsayes K.M., Lubner M.G., Shehata M.A., Fowler K., Kaoud A., Pickhardt P.J. (2024). Metabolic syndrome: Imaging features and clinical outcomes. Br. J. Radiol..

[3] Saklayen M.G. (2018). The Global Epidemic of the Metabolic Syndrome. Curr. Hypertens. Rep..

[4] Wilson P.W., D’Agostino R.B., Parise H., Sullivan L., Meigs J.B. (2005). Metabolic syndrome as a precursor of cardiovascular disease and type 2 diabetes mellitus. Circulation.

[5] Grundy S.M. (2016). Metabolic syndrome update. Trends Cardiovasc. Med..

[6] Vesa C.M., Zaha D.C., Bungău S.G. (2024). Molecular Mechanisms of Metabolic Syndrome. Int. J. Mol. Sci..

[7] Scurt F.G., Ganz M.J., Herzog C., Bose K., Mertens P.R., Chatzikyrkou C. (2024). Association of metabolic syndrome and chronic kidney disease. Obes. Rev..

[8] Tirandi A., Carbone F., Montecucco F., Liberale L. (2022). The role of metabolic syndrome in sudden cardiac death risk: Recent evidence and future directions. Eur. J. Clin. Investig..

[9] Protasiewicz-Timofticiuc D.C., Bădescu D., Moța M., Ștefan A.G., Mitrea A., Clenciu D., Efrem I.C., Roșu M.M., Vladu B.E., Gheonea T.C. (2024). Back to Roots: Dysbiosis, Obesity, Metabolic Syndrome, Type 2 Diabetes Mellitus, and Obstructive Sleep Apnea-Is There an Objective Connection? A Narrative Review. Nutrients.

[10] Kurnool S., McCowen K.C., Bernstein N.A., Malhotra A. (2023). Sleep Apnea, Obesity, and Diabetes—An Intertwined Trio. Curr. Diab. Rep..

[11] Nolan P.B., Carrick-Ranson G., Stinear J.W., Reading S.A., Dalleck L.C. (2017). Prevalence of metabolic syndrome and metabolic syndrome components in young adults: A pooled analysis. Prev. Med. Rep..

[12] O’Neill S., O’Driscoll L. (2015). Metabolic syndrome: A closer look at the growing epidemic and its associated pathologies. Obes. Rev..

[13] Reaven G.M. (1988). Banting lecture 1988. Role of insulin resistance in human disease. Diabetes.

[14] Kaplan N.M. (1989). The deadly quartet. Upper-body obesity, glucose intolerance, hypertriglyceridemia, and hypertension. Arch. Intern. Med..

[15] Alberti K.G., Eckel R.H., Grundy S.M., Zimmet P.Z., Cleeman J.I., Donato K.A., Fruchart J.C., James W.P., Loria C.M., Smith S.C. (2009). Harmonizing the metabolic syndrome: A joint interim statement of the International Diabetes Federation Task Force on Epidemiology and Prevention; National Heart, Lung, and Blood Institute; American Heart Association; World Heart Federation; International Atherosclerosis Society; and International Association for the Study of Obesity. Circulation.

[16] Zhang W., Chen C., Li M., Yan G., Tang C. (2024). Sex Differences in the Associations among Insulin Resistance Indexes with Metabolic Syndrome: A Large Cross-Sectional Study. Int. J. Endocrinol..

[17] Paniagua J.A. (2016). Nutrition, insulin resistance and dysfunctional adipose tissue determine the different components of metabolic syndrome. World J. Diabetes.

[18] Dichi I., Simão A.N., Vannucchi H., Curi R., Calder P.C. (2012). Metabolic syndrome: Epidemiology, pathophysiology, and nutrition intervention. J. Nutr. Metab..

[19] Noubiap J.J., Nansseu J.R., Lontchi-Yimagou E., Nkeck J.R., Nyaga U.F., Ngouo A.T., Tounouga D.N., Tianyi F.L., Foka A.J., Ndoadoumgue A.L. (2022). Global, regional, and country estimates of metabolic syndrome burden in children and adolescents in 2020: A systematic review and modelling analysis. Lancet Child Adolesc. Health.

[20] Hirode G., Wong R.J. (2020). Trends in the Prevalence of Metabolic Syndrome in the United States, 2011-2016. JAMA.

[21] Popa S., Moţa M., Popa A., Moţa E., Serafinceanu C., Guja C., Catrinoiu D., Hâncu N., Lichiardopol R., Bala C. (2016). Prevalence of overweight/obesity, abdominal obesity and metabolic syndrome and atypical cardiometabolic phenotypes in the adult Romanian population: PREDATORR study. J. Endocrinol. Investig..

[22] Vasques A.C., Novaes F.S., de Oliveira Mda S., Souza J.R., Yamanaka A., Pareja J.C., Tambascia M.A., Saad M.J., Geloneze B. (2011). TyG index performs better than HOMA in a Brazilian population: A hyperglycemic clamp validated study. Diabetes Res. Clin. Pract..

[23] Zhang Y., Wang F., Tang J., Shen L., He J., Chen Y. (2024). Association of triglyceride glucose-related parameters with all-cause mortality and cardiovascular disease in NAFLD patients: NHANES 1999-2018. Cardiovasc. Diabetol..

[24] Mirr M., Skrypnik D., Bogdański P., Owecki M. (2021). Newly proposed insulin resistance indexes called TyG-NC and TyG-NHtR show efficacy in diagnosing the metabolic syndrome. J. Endocrinol. Investig..

[25] Bala C., Gheorghe-Fronea O., Pop D., Pop C., Caloian B., Comsa H., Bozan C., Matei C., Dorobantu M. (2019). The Association Between Six Surrogate Insulin Resistance Indexes and Hypertension: A Population-Based Study. Metab. Syndr. Relat. Disord..

[26] Efrem I.C., Moța M., Vladu I.M., Mitrea A., Clenciu D., Timofticiuc D.C.P., Diaconu I.-D., Turcu A., Crișan A.E., Geormăneanu C. (2022). A Study of Biomarkers Associated with Metabolic Dysfunction-Associated Fatty Liver Disease in Patients with Type 2 Diabetes. Diagnostics.

[27] Amzolini A.M., Forţofoiu M.C., Barău Abu-Alhija A., Vladu I.M., Clenciu D., Mitrea A., Forţofoiu M., Matei D., Enăchescu V., Predescu O.I. (2021). Triglyceride and glucose index: A useful tool for non-alcoholic liver disease assessed by liver biopsy in patients with metabolic syndrome?. Rom. J. Morphol. Embryol..

[28] Amzolini A.M., Forțofoiu M.-C., Alhija A.B., Vladu I.M., Clenciu D., Mitrea A., Forțofoiu M., Matei D., Diaconu M., Tudor M.S. (2022). Triglyceride and Glucose Index as a Screening Tool for Nonalcoholic Liver Disease in Patients with Metabolic Syndrome. J. Clin. Med..

[29] Raimi T.H., Dele-Ojo B.F., Dada S.A., Fadare J.O., Ajayi D.D., Ajayi E.A., Ajayi O.A. (2021). Triglyceride-Glucose Index and Related Parameters Predicted Metabolic Syndrome in Nigerians. Metab. Syndr. Relat. Disord..

[30] Khan S.H., Sobia F., Niazi N.K., Manzoor S.M., Fazal N., Ahmad F. (2018). Metabolic clustering of risk factors: Evaluation of Triglyceride-glucose index (TyG index) for evaluation of insulin resistance. Diabetol. Metab. Syndr..

[31] Lim J., Kim J., Koo S.H., Kwon G.C. (2019). Comparison of triglyceride glucose index, and related parameters to predict insulin resistance in Korean adults: An analysis of the 2007-2010 Korean National Health and Nutrition Examination Survey. PLoS ONE.

[32] Yu X., Wang L., Zhang W., Ming J., Jia A., Xu S., Li Q., Ji Q. (2019). Fasting triglycerides and glucose index is more suitable for the identification of metabolically unhealthy individuals in the Chinese adult population: A nationwide study. J. Diabetes Investig..

[33] Abbasi F., Reaven G.M. (2011). Comparison of two methods using plasma triglyceride concentration as a surrogate estimate of insulin action in nondiabetic subjects: Triglycerides × glucose versus triglyceride/high-density lipoprotein cholesterol. Metabolism.

[34] Liu X.Z., Fan J., Pan S.J. (2019). METS-IR, a novel simple insulin resistance indexes, is associated with hypertension in normal-weight Chinese adults. J. Clin. Hypertens..

[35] Nabipoorashrafi S.A., Seyedi S.A., Rabizadeh S., Ebrahimi M., Ranjbar S.A., Reyhan S.K., Meysamie A., Nakhjavani M., Esteghamati A. (2022). The accuracy of triglyceride-glucose (TyG) index for the screening of metabolic syndrome in adults: A systematic review and meta-analysis. Nutr. Metab. Cardiovasc. Dis..

[36] Wan H., Cao H., Ning P. (2024). Superiority of the triglyceride glucose index over the homeostasis model in predicting metabolic syndrome based on NHANES data analysis. Sci. Rep..

[37] Son D.H., Lee H.S., Lee Y.J., Lee J.H., Han J.H. (2022). Comparison of triglyceride-glucose index and HOMA-IR for predicting prevalence and incidence of metabolic syndrome. Nutr. Metab. Cardiovasc. Dis..

[38] Mansoori A., Nosrati M., Dorchin M., Mohammadyari F., Derakhshan-Nezhad E., Ferns G., Esmaily H., Ghayour-Mobarhan M. (2025). A novel index for diagnosis of type 2 diabetes mellitus: Cholesterol, High density lipoprotein, and Glucose (CHG) index. J. Diabetes Investig..

[39] Laohabut I., Udol K., Phisalprapa P., Srivanichakorn W., Chaisathaphol T., Washirasaksiri C., Sitasuwan T., Chouriyagune C., Auesomwang C. (2020). Neck circumference as a predictor of metabolic syndrome: A cross-sectional study. Prim. Care Diabetes.

[40] Yang G.R., Dye T.D., Zand M.S., Fogg T.T., Yuan S.Y., Yang J.K., Li D. (2019). Association Between Neck Circumference and Coronary Heart Disease: A Meta-analysis. Asian Pac. Isl. Nurs. J..

[41] Bello-Chavolla O.Y., Almeda-Valdes P., Gomez-Velasco D., Viveros-Ruiz T., Cruz-Bautista I., Romo-Romo A., Sánchez-Lázaro D., Meza-Oviedo D., Vargas-Vázquez A., Campos O.A. (2018). METS-IR, a novel score to evaluate insulin sensitivity, is predictive of visceral adiposity and incident type 2 diabetes. Eur. J. Endocrinol..

[42] Widjaja N.A., Irawan R., Hanindita M.H., Ugrasena I., Handajani R. (2023). METS-IR vs. HOMA-AD and Metabolic Syndrome in Obese Adolescents. J. Med. Investig..

[43] Matsubayashi Y., Fujihara K., Yamada-Harada M., Mitsuma Y., Sato T., Yaguchi Y., Osawa T., Yamamoto M., Kitazawa M., Yamada T. (2022). Impact of metabolic syndrome and metabolic dysfunction-associated fatty liver disease on cardiovascular risk by the presence or absence of type 2 diabetes and according to sex. Cardiovasc. Diabetol..

[44] Eslam M., Newsome P.N., Sarin S.K., Anstee Q.M., Targher G., Romero-Gomez M., Zelber-Sagi S., Wai-Sun Wong V., Dufour J.F., Schattenberg J.M. (2020). A new definition for metabolic dysfunction-associated fatty liver disease: An international expert consensus statement. J. Hepatol..

[45] Godoy-Matos A.F., Silva Júnior W.S., Valerio C.M. (2020). NAFLD as a continuum: From obesity to metabolic syndrome and diabetes. Diabetol. Metab. Syndr..

[46] Leoni S., Tovoli F., Napoli L., Serio I., Ferri S., Bolondi L. (2018). Current guidelines for the management of non-alcoholic fatty liver disease: A systematic review with comparative analysis. World J. Gastroenterol..

[47] Kouvari M., Chrysohoou C., Skoumas J., Pitsavos C., Panagiotakos D.B., Mantzoros C.S., ATTICA study Investigators (2022). The presence of NAFLD influences the transition of metabolically healthy to metabolically unhealthy obesity and the ten-year cardiovascular disease risk: A population-based cohort study. Metabolism.

[48] Mota M., Popa S.G., Mota E., Mitrea A., Catrinoiu D., Cheta D.M., Guja C., Hancu N., Ionescu-Tirgoviste C., Lichiardopol R. (2016). Prevalence of diabetes mellitus and prediabetes in the adult Romanian population: PREDATORR study. J. Diabetes.

[49] World Health Organization (2008). Waist circumference and waist–hip ratio. Proceedings of the Report of a WHO Expert Consultation.

[50] Weststrate J.A., Deurenberg P. (1989). Body composition in children: Proposal for a method for calculating body fat percentage from total body density or skinfold-thickness measurements. Am. J. Clin. Nutr..

[51] Matthews D.R., Hosker J.P., Rudenski A.S., Naylor B.A., Treacher D.F., Turner R.C. (1985). Homeostasis model assessment: Insulin resistance and beta-cell function from fasting plasma glucose and insulin concentrations in man. Diabetologia.

